# Statement of Retraction: The relationship between hemodialysis mortality and the Chinese medical insurance type

**DOI:** 10.1080/0886022X.2019.1679426

**Published:** 2019-10-28

We, the Editor and Publishers of *Renal Failure*, have retracted the following article:

Xi Yao, Shaohua Chen, Wenhua Lei, Nan Shi, Weiqiang Lin, Xiaoying Du, Ping Zhang & Jianghua Chen, The relationship between hemodialysis mortality and the Chinese medical insurance type, *Renal Failure*, 41:1, 2019, 742–749, https://doi.org/10.1080/0886022X.2019.1652648.

The above article has been retracted due to Publisher error as it is an earlier version of an article that was ultimately accepted and published in *Renal Failure*.

The retracted article will remain online to maintain the scholarly record, but it will be digitally watermarked on each page as “Retracted”.

